# Technoethics in real life: AI as a core clinical competency

**DOI:** 10.1186/s44158-025-00233-2

**Published:** 2025-03-01

**Authors:** Elena Giovanna Bignami, Michele Russo, Federico Semeraro, Valentina Bellini

**Affiliations:** 1 Anesthesiology, Critical Care and Pain Medicine Division, Department of Medicine and Surgery, University of Parma, Parma, Italy; 2 Department of Anaesthesia, Intensive Care and Prehospital Emergency, Maggiore Hospital Carlo Alberto Pizzardi, Bologna, Italy

**Keywords:** Technoethics, Artificial Intelligence in Healthcare, Digital Literacy, Medical Education, Clinical Artificial Intelligence Skills, Intelligent Time, Ethics in Artificial Intelligence, Artificial Intelligence Integration, Physicianeers, Regulatory Frameworks

We are writing to highlight the urgent need to integrate technoethics into medical education and continuous professional development, particularly in the acquisition and maintenance of clinical artificial intelligence (AI) skills among healthcare personnel. The rapid advancements in AI within the field of medicine require a shift in perspective: AI-related competencies should extend beyond technical experts and be recognized as essential clinical skills for all healthcare providers.

Technoetics is a term coined by Roy Ascott, a British artist and theorist, that refers to the intersection of technology and consciousness. It explores how digital media, cybernetics, artificial intelligence, and immersive technologies (such as virtual reality, augmented reality, and telepresence) influence human perception, cognition, and creativity [1]. In the context of healthcare, this means ensuring that AI is designed and deployed in ways that promote equity, transparency, and patient-centered care while safeguarding against biases, ethical risks, and unintended harm. AI should not only complement but also enhance human decision-making, fostering a healthcare system where technological progress aligns with fundamental ethical responsibilities. The ethical integration of AI into clinical practice demands that healthcare professionals receive adequate training in evaluating AI outputs critically and responsibly, ensuring that they remain active stewards of patient welfare rather than passive adopters of automated recommendations.

As Pettigrew et al. articulate in their concept of "Physicianeers," the future of medicine requires clinicians to bridge engineering and medical sciences, ensuring that technological advancements are seamlessly incorporated into patient care [2]. However, this vision cannot be realized without a foundational emphasis on digital literacy underscoring the necessity for structured educational programs that equip medical professionals with the ability to critically assess, interpret, and apply AI-driven insights in their practice [3].

Moreover, the ethical and regulatory frameworks must evolve to support responsible AI adoption [4]. One crucial aspect of this evolution is ensuring that AI literacy is embedded within medical curricula and sustained through lifelong learning initiatives. This approach not only mitigates risks associated with AI deployment but also empowers clinicians to harness these tools effectively and ethically.

Beyond mere proficiency, the ethical implications of AI in medicine demand a framework that emphasizes responsibility, transparency, and accountability. Technoethics must guide the integration of AI into medical practice to ensure that algorithmic decision-making remains a tool to enhance, rather than replace, human clinical judgment. AI should be developed and applied in ways that uphold patient autonomy, equity in healthcare access, and the fundamental principles of medical ethics. Clinicians must be equipped not only with technical knowledge but also with the ability to critically evaluate AI recommendations and maintain their role as the ultimate decision-makers in patient care.

An essential component of this transformation is the concept of "Intelligent Time," where generative AI can be leveraged to automate bureaucratic and administrative tasks [5]. By offloading time-consuming documentation and data processing to AI systems, healthcare professionals can redirect their focus toward patient care, critical decision-making, and human-centered aspects of medicine. This paradigm shift has the potential to alleviate burnout and enhance the quality of care delivered in high-pressure environments such as anesthesia and critical care. However, effective implementation requires ensuring that AI-driven automation remains a support tool rather than an additional burden that demands excessive oversight from already overworked professionals.

Furthermore, the European TRAIN Initiative, as discussed by van Genderen et al. [6], emphasizes the necessity of human-centered and trustworthy AI within healthcare. The EU AI Act highlights the importance of regulatory frameworks that ensure transparency, accountability, and robustness in AI applications. The initiative's approach to responsible AI implementation demonstrates the need for standardized training programs that align with evolving regulations and provide healthcare institutions with the tools necessary for ethical AI integration.

Bowness et al. further highlight the growing necessity of medical leadership in AI development to ensure its meaningful integration into clinical practice. They argue that the medical community must actively engage in shaping AI applications to align with patient care needs rather than leaving these decisions solely to industry leaders. This reinforces the importance of embedding AI education into medical curricula to cultivate a generation of clinicians who are not only technologically proficient but also capable of guiding AI implementation in an ethical and clinically relevant manner [7].

Additionally, You and colleagues propose a clinical trials-informed framework for real-world AI implementation in healthcare. They emphasize the importance of a phased approach—covering safety, efficacy, effectiveness, and monitoring—to ensure that AI tools undergo rigorous validation before integration into clinical practice. This structured methodology aligns with the principles of technoethics by prioritizing patient safety, transparency, and equity while fostering a culture of responsible AI deployment in medicine [8].

Therefore, we urge academic institutions, professional societies, and healthcare organizations to recognize AI proficiency as a core clinical competency. The integration of AI-focused training must extend beyond medical school, fostering a culture of continuous learning that ensures clinicians remain adept at utilizing evolving technologies while upholding ethical standards. AI education should not be an isolated subject but rather an integrated part of medical training, seamlessly incorporated into clinical scenarios, case-based learning, and practical applications.

The future of medicine will be defined by the symbiotic relationship between human expertise and AI capabilities. By embracing technoethics as a guiding principle, we can ensure that AI serves as a catalyst for improving healthcare outcomes while reinforcing the irreplaceable role of human judgment, empathy, and ethical responsibility in medical practice. Figure 1 highlights crucial themes such as Technoethics, Intelligent Time, Clinical Skills, AI in Critical Care, Digital Literacy, and Medical Training, emphasizing the ethical and practical considerations necessary for responsible AI adoption in healthcare. The prominence of terms like Physicianeers, AI Proficiency, and Ethics in AI underscores the need for structured training programs that align with the evolving role of artificial intelligence in medicine.

**Fig. 1:**
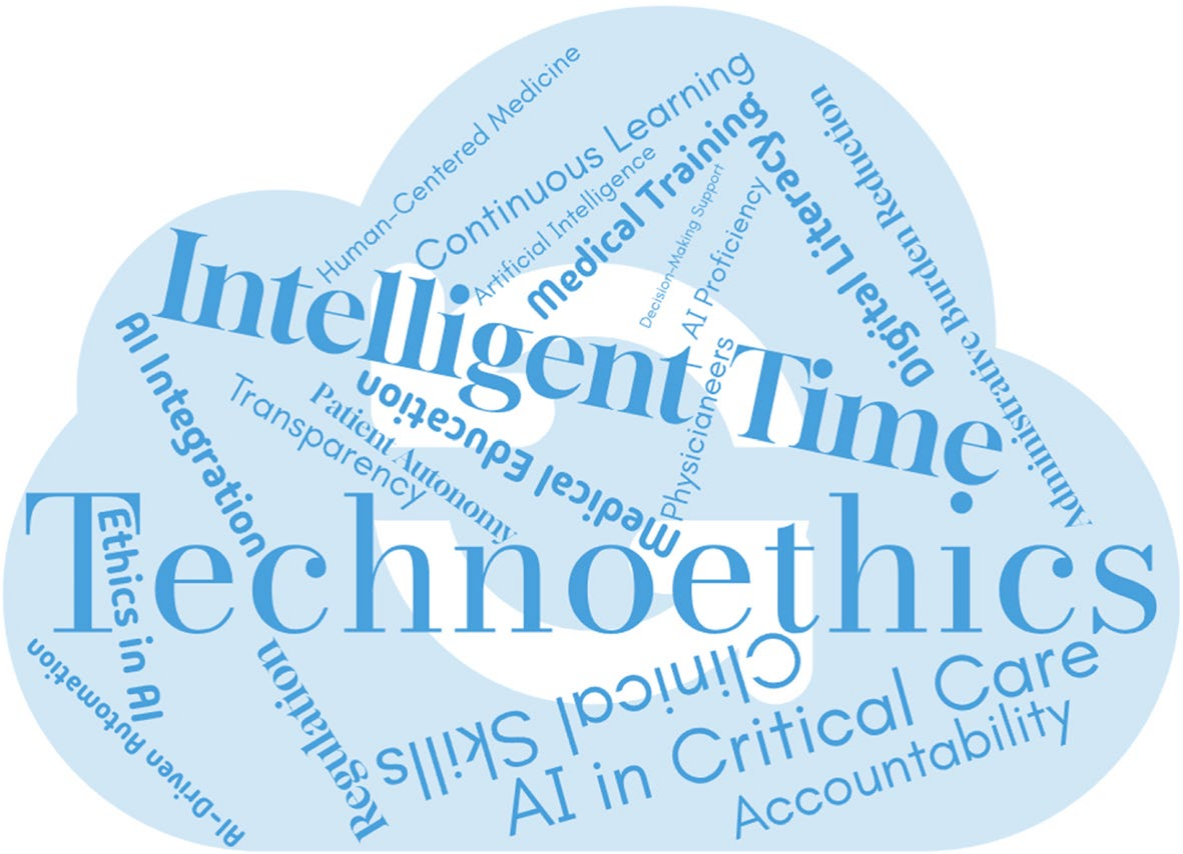
A word cloud representing key concepts in the integration of technoethics into medical education and clinical practice

## Data Availability

Not applicable. No datasets were generated or analysed during the current study.

## Abbreviations

AI: Artificial Intelligence
EU: European Union
TRAIN: Trustworthy AI Integration Initiative
